# Reverse Takotsubo Cardiomyopathy During Immediate Post-partum: A Case Report

**DOI:** 10.7759/cureus.36700

**Published:** 2023-03-26

**Authors:** David Jacobo Sanchez-Amaya, Miguel-Angel Lopez-Lizarraga, Mateo Gutierrez Castañeda, Diego Araiza-Garaygordobil, Alexandra Arias-Mendoza

**Affiliations:** 1 Cardiology, Instituto Nacional de Cardiología, Mexico City, MEX; 2 Coronary Care Unit, Instituto Nacional de Cardiología, Mexico City, MEX

**Keywords:** reverse takotsubo cardiomyopathy, acute cardiogenic pulmonary edema, acute systolic heart failure, cesarean birth, stress-related cardiomyopathy

## Abstract

Takotsubo cardiomyopathy or stress-induced cardiomyopathy is a particular entity with a transient left ventricular dysfunction without significant coronary artery obstruction, preceded by a stressful circumstance. Clinical presentation may mimic myocardial infarction, acute heart failure among the most common conditions. If suspected, the integration of clinical aspects, imaging results, and laboratory tests allows its diagnosis and proper management. Once described as a post-menopausal women's disease, is now recognized as a more frequent condition of young women, especially after stressful conditions such as post-surgical status and peripartum period, rendering as a disease with a certain predisposition to female patients, with a not always benign evolution. This case remarks an atypical presentation experiencing a first nigh fatal evolution but a later satisfactory recuperation.

## Introduction

Takotsubo cardiomyopathy or broken heart syndrome is triggered by emotional or physical stress [1,2]. It is defined as a reversible myocardial dysfunction along segmental wall motion alterations [1]. First reported in Japan, it has gained worldwide interest since its diagnosis is more prevalent. Typical description reckons four subtypes (midventricular, focal, reversed, and apical ballooning) being the latest and the most common ones; reversed or inverted subtype is a rare presentation. The following case shows an uncommon but clearly recognized variant in a patient in the immediate post-cesarean puerperium, presenting with significant hemodynamic deterioration and subsequent satisfactory recovery.

## Case presentation

**Figure 1:**
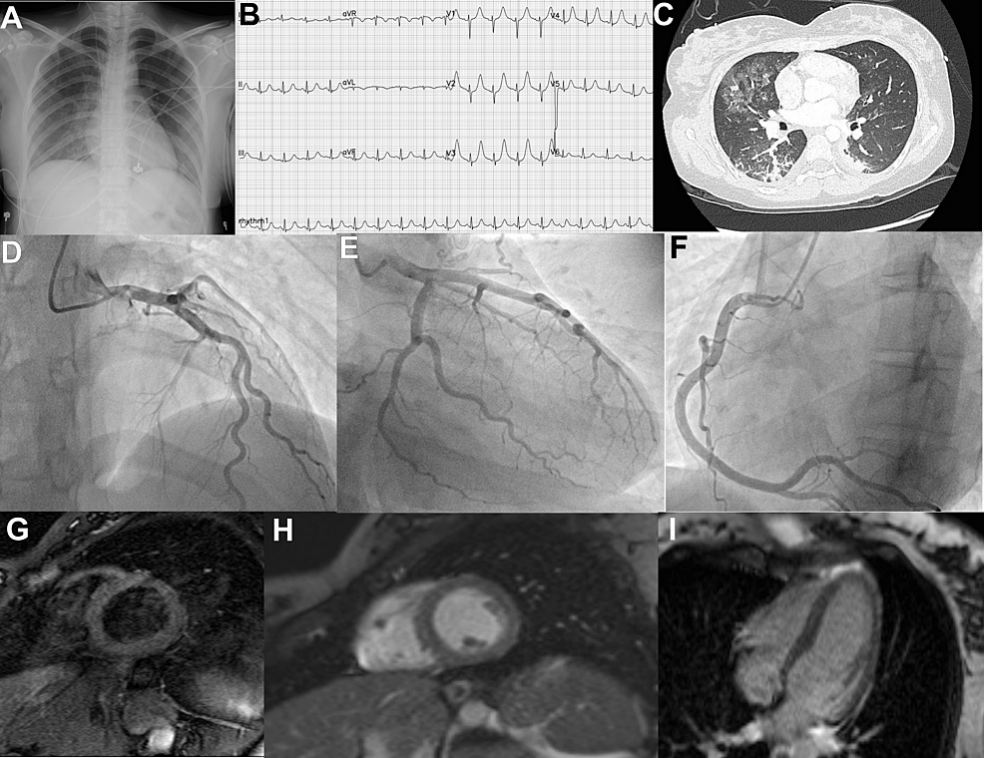
Graphic representation of reverse Takotsubo cardiomyopathy (rTCM) imaging studies A: Chest X-ray B: 12 leads electrocardiogram C: Chest CT noncontrast, axial view D-F: Coronary angiography G: T2-weighted sequence, short axis, mid view H: Delayed gadolinium enhancement inversion recovery sequence, short axis, basal view I: Delayed gadolinium enhancement inversion recovery sequence, 4 Chamber view

A young Hispanic and otherwise healthy woman in her twenties was admitted to elective cesarean delivery after a 38-week non-complicated pregnancy. No remarkable data was recollected upon anamnesis or physical examination, except for bicornuate uterus diagnosis in her first pregnancy. During immediate post-surgical care, she unexpectedly developed acute hypoxic respiratory failure and shock requiring mechanical ventilation and vasopressor support. Clinical exploration revealed bilateral rales consistent with Kerley B lines observed in chest roentgenogram (Figure 1A). 12 leads electrocardiogram showed diffuse hyperacute T waves and QTc (Figure 1B). Laboratory test found elevated myocardial injury, with a disproportionated NT-proBNP over troponin levels relationship (6216/293, upper reference level 201 pg/mL and 14 ng/mL, respectively) along anemia, 9.7 g/dL and no more remarkable data. The patient was transferred to a tertiary hospital center, where supportive care and diagnostic approach continued. Point of care ultrasonography exhibited B lines in both lungs and hyperdynamic cardiac contraction (Video 1), no further data could be gathered.

**Video 1:** Transthoracic echocardiography, Parasternal long axis view

Although D-dimer was among normal levels (0.4 µg/mL) pulmonary angiotomography scan was done since abrupt deterioration. The test showed no direct nor indirect signs of acute thromboembolic disease. However, both bilateral septal thickening and bilateral pleural effusion were described (Figure 1C). IV loop diuretic therapy achieved proper diuresis and during the first 24 hours after arrival, successful weaning ventilation took place along with pharmacological support withdrawal. Therefore, a diagnostic workup went on, including coronary angiography with no abnormality described (Figure 1D-1F). Cardiac magnetic resonance found basal and medium hypokinesis with hypercontractile apical segments (Video 2 and Video 3), mild systolic dysfunction, left ventricular ejection fraction (LVEF) 46%, along with edema in T2-weighted sequences in mid-septum, where also intramyocardial late gadolinium inversion recovery sequence was found (Figure 1G-1I), suggestive of Takotsubo syndrome.

**Video 2:** Cardiac magnetic resonance, 4 chambers view

**Video 3:** Cardiac magnetic resonance, cine 2 chambers view

Even though the delayed enhancement pattern was confined to only the mid-septum, taking into account the fact that the patient had a triggering factor, retrospectively an InterTAK scale score of 56 was calculated which provides a specificity above 95% for stress cardiomyopathy, thus integrating this diagnosis. Angiotensin inhibitor enzyme along with beta-adrenergic receptor blocker therapy administration ensued. After successful clinical recovery, the patient was dispatched to continue ambulatory monitoring.

## Discussion

Takotsubo cardiomyopathy (TCM) or Takotsubo syndrome (TTS) is a condition generally triggered by an emotional or physical stressor, characterized by a transient regional left ventricular (LV) systolic dysfunction. First described over 30 years ago in Japan by Hikaru Sato [1], the term is taken from the Japanese word referring to octopus trap due to the characteristic apical ballooning appearance [2]. Approximately 2% of all acute coronary-like syndromes (ACS) are ultimately diagnosed as TCM [3,4]. TCM usually occurs predominantly in older adults, mostly post-menopausal women [2,4,5], however, a growing number of reported cases are emerging in peripartum women [6], especially after cesarean delivery [2,4], like our case.

TCM has a not completely understood pathophysiology, but several mechanisms have been proposed that result in direct and indirect myocardial damage [2]. Among those, catecholamine-induced cardiotoxicity, both endogenous and exogenous sources, is the most accepted explication, altogether with microvascular dysfunction, and coronary vasospasm, hence leading to supply-demand mismatch and eventual reactive oxidative species overproduction and mitochondrial dysfunction, leading to myocardial stunning and electrophysiological derangement phenomena [2,3,5,7,8], with a characteristic reversible nature.

TCM is usually preceded by an identifiable acute stressor, either emotional or physical or a combination of both. Among 1759 patients of the International Takotsubo (InterTAK) Registry, 36% had a physical trigger, 28% an emotional trigger, 8% both types, and up to one-third had no identifiable cause [2]. A recent prospective study found 52% of cases were related to physical triggers, 32% to emotional triggers, and 17% to no identifiable circumstance [9]. The association between postpartum and TCM has been clearly established, especially after C-section surgery [4,10]; in a previous report of 15 women diagnosed with TTS (mean age 34 years) and no history of cardiomyopathy, who presented during the postpartum period, apical ballooning was documented in nine cases, midventricular involvement with apical preservation in five cases, and basal ballooning in a single case [10]. Emotional triggers have been associated with a favorable prognosis compared to physical situations [2,7]. However, as could be observed, the mid-term evolution in our case was favorable.

In a meta-analysis done by Singh et al. [11], that included a total of 1664 patients with TTS, 48% had an initial presentation mimicking non-ST elevation myocardial infarction (NSTEMI), 40% had at least moderate systolic dysfunction and 21% developed acute pulmonary edema with an average length of recuperation of 6.4 days, similar to our case.

Clinical manifestations range from mild presentation to a near-fatal condition. In the majority of cases, patients refer chest pain and other manifestations typical of ACS, however, our patient only referred rapidly progressive dyspnea. Most patients have experienced a stressful event in the previous 1-5 days [12], which correlates to our case in which the stressful event occurred just one day earlier. Characteristic findings on the ECG include QTc interval prolongation (average 542 ms) in up to 50% of patients [2,13], as well as ST segment elevation in derivation aVR without the presence of pathological Q waves. The latter has a sensitivity of up to 91% and a specificity of 96% [2,4]. Only QT interval prolongation was found in this case, which is related to ventricular arrhythmias and the risk of sudden cardiac death [14], but fortunately, this did not occur.

Cardiac biomarkers are always elevated, reflecting myocardial acute inflammation and perhaps even some necrosis. Troponin values usually peak within 24 hours and natriuretic peptide just after 48 hours. Khan et al. conducted a meta-analysis about the issue, which found an elevated natriuretic peptide/troponin ratio [15], compatible with our case presentation [2]. Wall motion abnormalities detection is achieved through echocardiographic evaluation, which has become the first method of choice. Since TCM by definition implies regional or global motion abnormalities, a myriad of complications may occur. Left ventricle outflow tract obstruction (LVOTO) appears in up to 20% of cases. Less common, cardiogenic shock, mitral regurgitation, intracavitary thrombi formation, stroke, and ventricular rupture have been reported [2,14].

Apical ballooning with basal hypercontractility is found in up to 82% of cases. Other variants like mid-ventricular, basal, and focal wall abnormalities accounted for 15%, 2%, and 1%, respectively [2]. Reverse (basal akinesis) Takotsubo cardiomyopathy (rTCM) patients tend to be younger with a mean age of 30 years [4] and more prone to pulmonary edema and cardiogenic shock [3,8] in accordance with patients’ presentation. Furthermore, they tend to have more ST depression, longer QTc values [7], and higher levels of cardiac biomarkers [3,5,16].

TCM diagnosis usually takes place just after ACS is ruled out. Abe et al. [16], in an effort towards facilitating a diagnosis approach, introduced the first set of diagnostic criteria in 2003. Thenceforth, several diagnostic criteria have been developed - the Revised Mayo Clinic criteria, 2008 and InterTAK criteria, 2018 the most used [2,14]. Other imaging techniques include cardiac magnetic resonance (CMR), single-photon emission computer tomography (SPECT), and positron emission tomography (PET) among others. CMR is a very useful imaging modality, rendering information about temporal evolution and allowing the diagnosis of the majority of cases. Typical findings are reversible myocardial inflammation and edema, distinguished upon T2 weighted sequences and T2 native mapping. CMR also accurately defines segmental motion abnormalities and through inversion recovery sequence with late gadolinium enhancement, the presence of edema or myocardial fibrosis [2,9]. In our case, we observed an unusual pattern of late gadolinium, just confined to the mid-septum. However, the diagnosis was supported by the InterTAK Diagnostic Score, which yielded a score of 56 points. Using a cut-off value of 40 points, the sensitivity for the presence of TTS was 89% (up to 94.7% for a score value of ≥50), and the specificity was 91% [17].

Initial management depends on the clinical syndrome severity. A mild disease might go underdiagnosed. If ACS-like syndrome or acute heart failure occurs, supportive therapy is mandatory and if tolerated, neurohormonal blockade should be instated. No randomized trial so far had taken place in order to prove any therapeutic approach, however, angiotensin enzyme inhibitor (ACEI) and angiotensin receptor blockers (ARB) along with Beta-blockers have been a mainstay in mid and long-term management [2,12]. Despite conflictive data, most studies have shown ACEI/ARB reduction of recurrence, but not betablockade therapy [2,8,14,18]. However, these pharmacological therapies are the cornerstone of medical management, extending up to three months or until LV dysfunction improves [8,14].

TCM in-hospital mortality is lower compared to acute myocardial infarction one. Following medical discharge, 95% of cases will experience full recovery after 3-6 months [12], and just a small percentage relapse throughout the time; its risk is about 2% per year during the first four years and then decreases to 1% yearly later [7].

## Conclusions

This case represents an atypical Takotsubo cardiomyopathy in an otherwise healthy woman in a post-cesarean delivery setting, complicated with acute pulmonary edema and hemodynamic collapse. No other alterations were found but transitory ventricular dysfunction and focal late gadolinium, however, diagnostic criteria application allowed proper diagnosis and treatment, consistent with the neurohormonal blockade, that ensued leading to successful recovery.
